# Methicillin-resistant *Staphylococcus aureus*-associated orbital cellulitis: a case series

**DOI:** 10.1007/s10792-023-02698-y

**Published:** 2023-04-07

**Authors:** Terence Ang, Cassie Cameron, Jessica Y. Tong, Geoff Wilcsek, Jeremy Tan, Sandy Patel, Dinesh Selva

**Affiliations:** 1 Discipline of Ophthalmology and Visual Sciences, The University of Adelaide, Adelaide, SA 5000 Australia; 2 Department of Ophthalmology, The Royal Adelaide Hospital, Adelaide, SA Australia; 3 Department of Ophthalmology, Sydney Children’s Hospital, Randwick, NSW Australia; 4 Department of Medical Imaging, Royal Adelaide Hospital, Adelaide, SA Australia

**Keywords:** Orbital cellulitis, Methicillin-resistant staphylococcus aureus, MRSA, Orbital, Infection

## Abstract

**Purpose:**

In recent years, methicillin-resistant *Staphylococcus aureus* (MRSA) orbital cellulitis (OC) has drawn increasing clinical and public health concern. We present a case series of MRSA OC encountered at four Australian tertiary institutions.

**Methods:**

A multi-centre retrospective case series investigating MRSA OC in Australia from 2013 to 2022. Patients of all ages were included.

**Results:**

Nine cases of culture-positive non-multi-resistant MRSA (nmMRSA) OC were identified at four tertiary institutions across Australia (7 male, 2 female). Mean age was 17.1 ± 16.7 years (range 13-days to 53-years), of which one was 13 days old, and all were immunocompetent. Eight (88.9%) patients had paranasal sinus disease, and seven (77.8%) had a subperiosteal abscess. Four (44.4%) had intracranial extension, including one (11.1%) case which was also complicated by superior sagittal sinus thrombosis. Empirical antibiotics, such as intravenous (IV) cefotaxime alone or IV ceftriaxone and flucloxacillin, were commenced. Following identification of nmMRSA, targeted therapy consisting of vancomycin and/or clindamycin was added. Nine (100%) patients underwent surgical intervention. Average hospital admission was 13.7 ± 6.9 days (range 3–25 days), with two patients requiring intensive care unit (ICU) admission due to complications related to their orbital infection. All patients had favourable prognosis, with preserved visual acuity and extraocular movements, following an average follow-up period of 4.6 months (range 2–9 months).

**Conclusion:**

NMMRSA OC can follow an aggressive clinical course causing severe orbital and intracranial complications across a wide demographic. However, early recognition, initiation of targeted antibiotics and surgical intervention when required can effectively manage these complications and achieve favourable visual outcomes.

## Introduction

Orbital cellulitis (OC) is a sight-threatening infection breaching the orbital septum and is characterised by periorbital oedema, erythema, fever, pain and proptosis, often presenting with concurrent paranasal sinusitis or recent orbital trauma [1, 2]. Complications can be severe and life-threatening, including optic neuropathy, subperiosteal or intracranial abscesses, meningoencephalitis, cavernous sinus thrombosis and sepsis [1]. Thus, emergent diagnosis and prompt treatment with antibiotics and/or surgical intervention reduces mortality and morbidity [1].

Bacterial OC most commonly originates from acute rhinosinusitis, with organisms implicated including *Staphylococcus aureus* and *Streptococcus pneumoniae.* Empirical antibiotics are guided by relevant epidemiology, along with clinical features and risk factors, such as immunocompromised [1]. Recently, methicillin-resistant *Staphylococcus aureus* (MRSA)-associated infections have drawn increasing clinical and public health concern in both developing and developed countries, leading to an increased use of vancomycin and combination antimicrobial therapy [3, 4]. Empirical antibiotics coverage for MRSA is guided by local epidemiology and clinical suspicion, such as those with critical illness [3].

We present a case series of 9 MRSA-related OC encountered at tertiary institutions across Australia. This study aims to characterise the clinico-radiological features and outcomes associated with MRSA OC.

## Methods

This was a multi-centre retrospective non-comparative case series investigating MRSA OC in Australia between 2013 and 2022. Inclusion criteria were patients of all ages with a clinico-radiological diagnosis of MRSA culture-positive OC. Pre-septal cellulitis (i.e. no clinico-radiological evidence of post-septal involvement) and microbiological isolates without MRSA were excluded.

Patients were identified from the Oculoplastics Unit at the Royal Adelaide Hospital (Adelaide, Australia), Flinders Medical Centre (Adelaide, Australia), Women’s and Children’s Hospital (Adelaide, Australia), Sydney Children’s Hospital (Randwick, Australia) and Sydney Eye Hospital (Sydney, Australia). Data collected included patient demographics (age, gender, past medical history and medications), clinical presentation, laboratory investigations (white cell count, C-reactive protein and microbiological analysis), radiological features, course of management and clinical outcomes.

Where applicable, results are expressed as means ± standard deviation (σ) and presented in relevant tables. Dimensions of lesions, as determined radiologically, are reported in the format of antero-posterior x transverse x coronal (millimetres). All research was conducted in accordance with the Declaration of Helsinki and was approved by our Institutional Review Board.

## Results

We present 9 cases (7 male, 2 females) of culture-positive non-multi-resistant MRSA (nmMRSA) OC at various tertiary institutions across Australia within a 10-year period from 2013 to 2022. This included 6 children and 3 adults, with a mean age of 17.1 ± 16.7 years (range 13 days to 53 years). The mean duration of symptoms prior to presentation was 2.8 ± 2.0 days (range 1–7 days). Relevant past medical history included chronic sinusitis (2/9, 22.2%) and asthma (1/9, 11.1%). Additionally, one (11.1%) had cocaine-induced nasal cavity necrosis with a perforated septum, and nasal hardware from a previous trauma. No patients were immunocompromised, and all were defined as nmMRSA infections. A summary of the cases is outlined in Table 1.

**Table 1:** Summary of clinical features, management and outcomes

Patient	Gender/age (years)	Relevant past medical history	Complications	Management	Admission (days)	Follow-up (Months)
1	M/19	Asthma	SPA, intracranial extension (frontal subdural empyema and meningitis)	ICU admission; IV vancomycin + FESS	8	2
2	M/35	Chronic sinusitis; previous MVA with metal hardware in cheek and nose	Pre-septal abscess	IV vancomycin and flucloxacillin (changed to meropenem) + incision and drainage of pre-septal abscess + FESS due to clinical deterioration	23	6
3	M/13 days	Nil	SPA	IV vancomycin and cefotaxime + examination under anaesthesia with marsupialisation of infected dacryocystocele	11	9
4	M/53	Chronic sinusitis	SPA; intracranial extension (frontal subdural empyema and meningitis)	IV ceftriaxone, vancomycin and metronidazole + FESS + septoplasty + SPA drainage via skin crease incision	14	4
5	F/8	Asthma; allergic rhinitis	SPA; frontal subdural empyema; SSS thrombosis; pneumonia; linezolid overdose	ICU admission; IV ceftriaxone, vancomycin and clindamycin + FESS + SPA drainage via superolateral brow incision + enoxaparin	25	8
6	M/9	Nil	Orbital abscess	IV antibiotics + orbital abscess drainage via lid crease incision	3	3
7	M/6	Nil	SPA	ICU admission; IV clindamycin + SPA drainage via canthotomy/cantholysis + FESS	15	3
8	F/13	Nil	SPA	IV vancomycin and cefotaxime, later stepped down to clindamycin; IV dexamethasone for 6 days with oral prednisolone taper; SPA drainage via skin crease incision + FESS	10	1.5
9	M/11	Autism Spectrum Disorder	SPA, sub-galeal abscess, epidural abscess and meningitis	IV vancomycin; SPA drainage via skin crease incision + bilateral FESS	14	LTF

*M* male, *F* female, *MVA* motor vehicle accident, *SPA* subperiosteal abscess, *SSS* superior sagittal sinus, *ICU* intensive care unit, *IV* intravenous, *FESS* functional endoscopic sinus surgery, *LTF* lost to follow-up

The most common clinical features included periorbital oedema (8/9, 88.9%), pain (8/9, 88.9%), proptosis (6/9, 66.7%) and restricted extraocular movements (6/9, 66.7%). Other common findings were globe dystopia (4/9, 44.4%), mechanical ptosis (4/9, 44.4%), fever (5/9, 55.5%), conjunctival hyperaemia (4/9, 44.4%) and ocular discharge/lacrimation (3/9, 33.3%). One (11.1%) patient had optic neuropathy, which resolved at final follow-up. Two (22.2%) had neurological symptoms including fluctuating consciousness and generalised tonic–clonic seizures (1/9, 11.1%) and right facial palsy (1/9, 11.1%) which was presumed to be secondary to parotid inflammation. Two (22.2%) had a preceding upper respiratory tract infection.

Laboratory investigations showed elevated C-reactive protein in 88.9% (mean CRP: 178.0 ± 100.9 mg/L) and leucocytosis in 88.8% (mean white cell count: 17.9 ± 5.2 × 10^9^/L). NMMRSA-positive cultures were isolated from a combination of sinus or orbital abscess swabs (6/9, 66.7%), nasal swabs (3/9, 33.3%) and blood cultures (1/9, 11.1%).

**Fig. 1:**
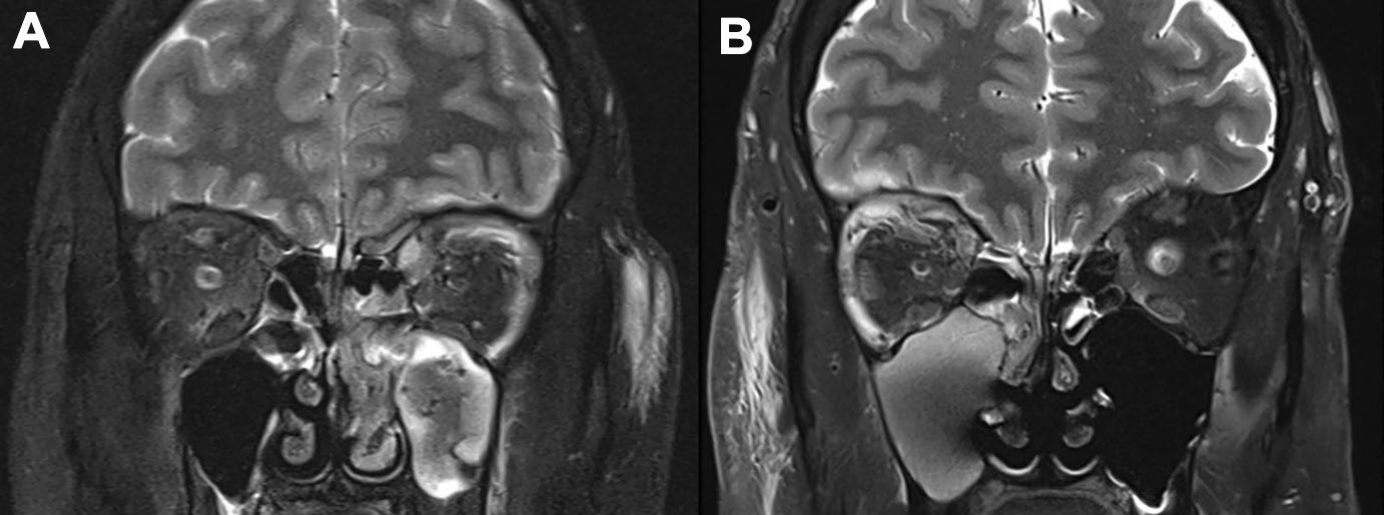
T2-weighted fat-suppressed MRI demonstrating heterogeneous hyperintensity in the region of concern. **A** and **B** demonstrate superolateral extraconal inflammatory changes in the left (Patient 1) and right orbits (Patient 4), respectively

**Fig. 2:**
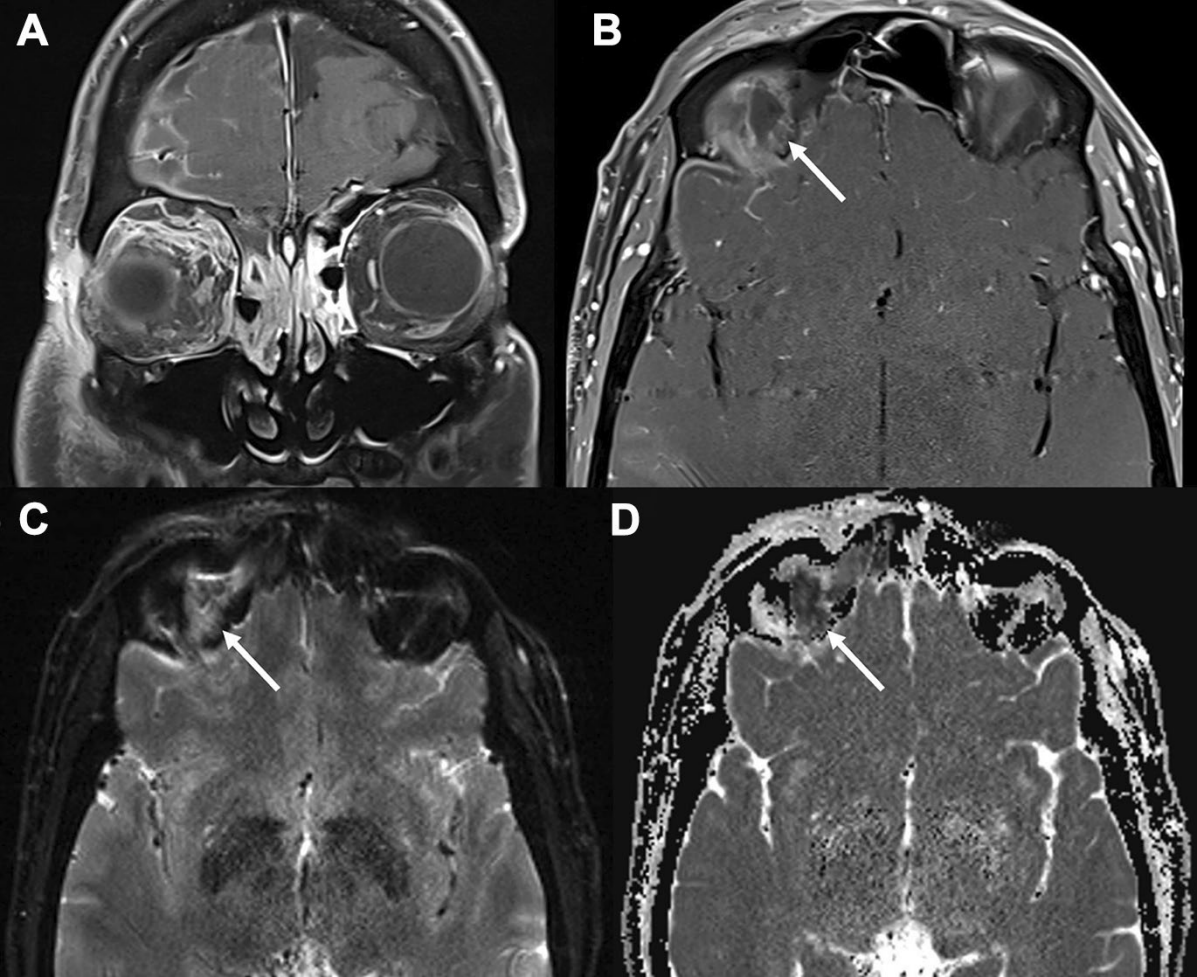
MR orbital imaging in Patient 4. **A** and **B** demonstrate T1 fat-suppressed contrast-enhanced MRI of the superior SPA (white arrow) with central non-enhancement indicative of an abscess. **C** and **D** demonstrate high signal (white arrow) on diffusion-weighted imaging and low signal on apparent diffusion coefficient (ADC) mapping exhibiting a low signal (white arrow), respectively, consistent with an abscess

All patients underwent contrast-enhanced computed tomography (CT), and four (44.4%) also had magnetic resonance imaging (MRI) due to clinical concern for intracranial complications. Table 2 summarises the radiological features encountered. On CT and MRI, all patients demonstrated pre-septal swelling and stranding of the extraconal and/or intraconal fat. Inflammatory changes within the orbit appeared as regions of heterogeneous hyperintensity on T2-weighted fat-suppressed sequences (Fig. 1A and B). One patient had a pre-septal abscess, four patients had lacrimal gland involvement, and five had EOM involvement. The superior ophthalmic vein and cavernous sinus were patent in all cases. Eight (88.9%) patients had paranasal sinus disease, and seven (77.8%) had subperiosteal abscesses (SPA). SPAs were located along the medial (2/7, 28.6%), superolateral (2/7, 28.6%), superomedial (1/7, 14.3%), superior (1/7, 14.3%) and inferior (1/7, 14.3%) orbital walls. One (11.1%) patient also had an extraconal orbital abscess, located laterally to the lateral rectus and contiguous but not directly involving the lacrimal gland (Patient 6). Abscess volumes ranged from 325 mm^3^ to 4620 mm^3^. Of the four patients who had an MRI, diffusion restriction on diffusion-weighted imaging (DWI) was observed in all the abscesses (Fig. 2). Four (44.4%) had intracranial extension with evidence of dural/leptomeningeal enhancement and frontal subdural collections, including one patient (11.1%) with a sub-galeal abscess with no osteomyelitis and multiple extradural empyemas over the right frontal and parasagittal regions (Patient 9), and one (11.1%) had a superior sagittal sinus thrombosis, which remained stable throughout admission (Patient 5).

**Table 2:** Summary of radiological features in MRSA OC cases

Patient	CT	MRI	Abscesses	Paranasal sinus	Additional features
1	Medial extraconal fat stranding	Heterogeneous hyperintense T2 signal in superior, superolateral and superomedial extraconal region with subtle intraconal involvement; lacrimal gland involvement; significant SO, MR and SMC involvement; optic sheath enhancement	SPA at superomedial orbital wall (16 × 4x6.5 mm)^#^	Mucosal disease in left ethmoid, frontal and maxillary	Pre-septal swelling, proptosis; dural enhancement of left frontal lobe; frontal subdural empyema
2	Medial extraconal fat stranding	Not conducted	Pre-septal abscess	Partial opacification of right frontal, bilateral ethmoid and maxillary	Pre-septal swelling; proptosis; antero-medial soft tissue phlegmon (27 × 8x28mm); air pockets in lacrimal sac
3	Intra- and extraconal fat stranding	Not conducted	SPA at medial orbital wall (27 × 2.87x6mm) causing displacement of MR	Mucosal thickening (13-days-old)	Pre-septal swelling; proptosis; lacrimal sac enlargement
4	Superior extraconal fat stranding	Heterogeneous hyperintense T2 signal in superior extraconal region; lacrimal gland involvement; SMC and LR involvement	SPA at superior orbital wall (13 × 5x5mm)^#^	Mucosal disease of ethmoid bilaterally, right frontal and maxillary	Pre-septal swelling; proptosis; leptomeningeal and dural enhancement; frontal subdural empyema
5	Superior extraconal fat stranding	Hyperintense T2 signal intra- and extraconal regions superolaterally*; lacrimal gland involvement; LR and SMC involvement; dural thickening and enhancement over right anterior frontal and temporal lobes	SPA at superolateral orbital wall (17.8 × 7.6x9mm)^#^	Mucosal thickening within the right maxillary, ethmoid and frontal	Pre-septal swelling; proptosis; SSS thrombosis; frontal subdural empyema
6	Extraconal and mild intraconal fat stranding	Not conducted	Orbital abscess in lateral orbit (24 × 14 mm) causing left globe dystopia	Clear	Pre-septal swelling; enlargement of LR; lacrimal gland enhancement
7	Extraconal fat stranding	Not conducted	SPA at inferior orbital floor causing superior displacement of IR (14 × 11x30)	Opacification of left maxillary, bilateral ethmoid and sphenoid sinuses and partial opacification of the left frontal sinus and right antrum	Pre-septal swelling; proptosis; subcutaneous nasal abscess and intranasal abscess
8	Antero-lateral intra- and extraconal fat stranding	Not conducted	SPA at medial orbital wall (22 × 10x16) causing displacement of MR	Mucosal thickening of maxillary, ethmoid and sphenoid sinuses	Pre-septal swelling
9	Superolateral and superomedial extraconal fat stranding	Heterogeneous hyperintense T2 signal in superior extraconal region; SMC involvement; right and left frontal dural enhancement	SPA at superolateral (11 × 10.6x5.3 mm) orbital wall causing displacement of the SMC	Opacification and mucosal thickening of paranasal sinuses (Right > Left)	Bilateral pre-septal swelling with sub-galeal abscess (19 × 5x28mm) over the para-midline frontal bone with no osteomyelitis; enlargement of SMC; three epidural empyemas over right frontal region, right parasagittal aspect of the planum sphenoidale and in the anterior sella turcica

Radiological dimensions have been reported as antero-posterior x transverse x coronal (mm).

*SMC* superior muscle complex, *SO* superior oblique, *MR* medial rectus, *IR* inferior rectus, *SPA* subperiosteal abscess, *SSS* superior sagittal sinus.

*Motion artefact resulted in poor image quality of orbits. ^#^All SPAs demonstrated diffusion restriction on MRI

Specific antibiotic and surgical management are outlined in Table 1. Empirical antibiotics, such as intravenous (IV) cefotaxime alone or IV ceftriaxone and flucloxacillin, were initially commenced*.* Nine (100%) patients underwent surgical intervention, including functional endoscopic sinus surgery (FESS) (8/9, 88.9%), SPA/orbital abscess drainage (6/9, 66.7%) and incision and drainage of a pre-septal abscess (1/9, 11.1%), which were performed alone or in combination. One (11.1%) patient underwent surgical exploration and marsupialisation of an infected dacryocele (Patient 3). The superior sagittal sinus thrombosis was treated with Enoxaparin, which was continued for three months post-discharge (Patient 5).

Average duration of admission was 13.7 ± 6.9 days (range 3–25 days). Three (33.3%) patients required Intensive Care Unit (ICU) admission, two for intracranial infection (Patients 1 and 5), while one ICU admission was unrelated to orbital infection and due to an anaphylactic reaction to codeine (Patient 7). Long-term sequelae of infection were observed including mild cognitive impairment at 4-month follow-up (Patient 4). On subsequent MRI, there was complete recanalisation of the superior sagittal sinus thrombosis following anticoagulation with no persisting neurological deficits (Patient 5). One patient had residual periorbital oedema at discharge, but full VA and EOM (Patient 9). The remaining patients had complete resolution of ocular symptoms consisting of preserved VA, no optic neuropathy or ptosis and full EOM following an average follow-up period of 4.6 months (range 2–9 months). One patient was lost to follow-up due to transfer of care to another tertiary institution upon discharge (Patient 9).

## Discussion

*Staphylococcus aureus* and *Streptococcus* species have been reported as the predominant pathogens in OC, often secondary to acute rhinosinusitis. However, other organisms include *Haemophilus influenzae*, *Moraxella catarrhalis* and anaerobes [1, 3, 5]. MRSA has emerged as an important public health concern, accounting for a significant proportion of soft tissue infections, including pre-septal cellulitis [5–10]. The incidence of MRSA OC varies within the literature, ranging from 21 to 73%, and is likely affected by the changing epidemiological trends of specific institutions and geographical locations [1]. In Australia (Adelaide, South Australia), the incidence of MRSA orbital cellulitis has been reported to be 28.6% between a 10-year period (2012 to 2022), with 50% of cases identified between 2021 and 2022 [11]. Previously, MRSA had represented 21.7% of all *Staphylococcus Aureus* of all ocular infections (Adelaide, South Australia); however, no cases of OC were included [12]. MRSA OC has also become prevalent in various geographical locations. For example, Shih et al. reported an increasing incidence of MRSA OC from 14.5% to 37.5% over two 10-year periods between 2000–2009 and 2010–2019 in Taiwan [6]. Meanwhile, Pandian et al. reported in 2011 that MRSA was the most common organism identified, responsible for 38% of culture-positive OC at their tertiary institution in India [10]. Though rationalised antibiotic management remains a central focus in the era of antibiotic resistance, early recognition of resistant organisms in OC is essential to prevent serious complications.

This series of nmMRSA OC demonstrates a fulminant disease course with cases of intracranial extension, superior sagittal sinus thrombosis and overall complicated/prolonged hospital admission. Nevertheless, early intervention with targeted antibiotics and/or surgical intervention reduced the risk of morbidity and mortality, with good prognosis observed in our cases.

Previous studies have reported differences in the clinical features between MRSA OC and other typical organisms [2, 5]. In 2012, Mathias et al. reported a case series of 15 MRSA OC cases, surmising that the following atypical features may raise suspicion of MRSA OC: absence of preceding upper respiratory tract infection; lacrimal gland focus; multiple orbital abscesses on imaging; or absence of adjacent paranasal sinus disease [5]. However, other studies and case reports have not validated these findings [2]. These features were also largely discordant with our findings: although only one patient had a preceding upper respiratory tract infection, two had chronic sinusitis, and nine (90%) patients had radiological evidence of paranasal sinus disease. Prior literature has also reported an association with a young age of presentation < 1 year old including immunocompetent individuals [2, 8, 9, 13]. Although there was a paediatric predominance (8/11, 72.7%) in our study, only 1 infant was aged < 1 year old. Ultimately, MRSA OC may have a diverse clinico-radiological presentation, and a broad and heterogeneous patient demographic may be affected.

MRSA OC was associated with a prolonged and complicated clinical course, and this has been demonstrated in prior studies of MRSA-associated ocular infections in South Australia [12]. The incidence of SPAs in bacterial OC ranges from 15–62% [14–18]. However, 81.8% of our patients developed a SPA, with 4 cases located in the superior orbit. In MRSA OC, SPAs and orbital abscesses may develop rapidly, and multiple abscesses may also form in other regions of the body in cases of septicaemia [8, 9]. Septicaemia occurred in 1 case which was also complicated by pneumonia (Patient 5); however, no cases had abscesses located in other regions of the body. Intracranial extension of infection and septic cerebral venous sinus thrombosis have significant morbidity and mortality, but the exact incidence is unknown [19, 20]. The cavernous sinus is most commonly affected, with sagittal sinus thrombosis involvement remaining very rare. (14) Intracranial extension was encountered in three (30%) cases, including one patient with a superior sagittal sinus thrombosis. *Staphylococcus aureus* reportedly accounts for 60–70% of all septic cerebral sinus thrombosis. (14) Several case reports have detailed cavernous sinus thrombosis secondary to MRSA-related orbital infections with high rates of mortality and morbidity, including blindness and impaired ocular motility [20–24]. The clinical course of MRSA infections has been more aggressive compared to other microbial causes [5, 10, 25]. Various factors may complicate management of MRSA infections including antimicrobial resistance and presence of highly virulent mechanisms including production of toxins, adhesion proteins and immune evasion [26]. For example, the Panton–Valentine leucocidin (PVL) toxin is associated with abscess formation [27]. Additionally, acquired phenotypic traits observed in recently emerging MRSA strains allow them to be more virulent and more effective at colonisation [26]. The incidence of profound visual loss in OC varies, with reportedly 3–27% of patients experiencing severe vision loss to the degree of light perception or worse [5]. Several patients in our series had prolonged hospital admissions with significant complications; however, full resolution of VA and motility with no cases of visual compromise or ocular sequelae were observed.

This study had several limitations due to its retrospective nature and MRSA OC remaining relatively uncommon. Additionally, detection of MRSA colonisation on less reliable culture methods (e.g. nasal swabs) may influence initial selection of empirical antibiotics, even if other pathogens are responsible for the orbital infection. Intraoperative swabs with direct access to the abscess improve the reliability and yield of microbiological cultures [28]. Thorough clinical assessment along with awareness of and systematic updates on the local epidemiology will help guide appropriate initiation of empirical nmMRSA active antibiotics when treating OC.

In conclusion, our case series presents the clinico-radiological presentations and outcomes of nmMRSA OC. This series demonstrates that nmMRSA OC can be associated with a complex disease course and the potential to develop severe orbital and intracranial complications, necessitating prolonged admission. Early initiation of appropriate antibiotic therapy and surgical intervention can achieve a favourable visual prognosis.
